# Treatment options for hypertrophic obstructive cardiomyopathy in the era of myosin inhibitors

**DOI:** 10.3389/fcvm.2026.1814860

**Published:** 2026-10-27

**Authors:** Chukwunenyem Nwuba, Jason N. Dungu

**Affiliations:** 1 Mid and South Essex NHS Foundation Trust, Basildon, Essex, United Kingdom; 2 Anglia Ruskin University School of Medicine and Medical Technical Research Centre, Chelmsford, Essex, United Kingdom

**Keywords:** aficamten, alcohol septal ablation, hypertrophic cardiomyopathy (HCM), hypertrophic obstructive cardiomyopathy, left ventricular outflow tract obstruction, mavacamten, myosin inhibitors, septal reduction therapy

## Abstract

Hypertrophic obstructive cardiomyopathy (HOCM) is characterised by left ventricular outflow tract obstruction (LVOTO) caused by septal hypertrophy and its interaction with the mitral valve and systolic anterior motion. Symptoms include dyspnoea, angina, palpitations, syncope, fatigue and sudden cardiac death. This review provides a comprehensive overview and breakdown of current available treatment options, including traditional medical therapies and surgical septal reduction therapies in the era of new emerging pharmacologic options, including myosin inhibitors. Traditional pharmacotherapy includes non-vasodilating beta blockers, calcium-channel blockers and disopyramide. For persistent symptoms, surgical myectomy and alcohol septal ablation remain important septal reduction therapies, with treatment choice depending on anatomy, procedural risk and site-specific expertise. Myosin inhibitors, including mavacamten and aficamten, offer a targeted approach to addressing the hypercontractility characteristic of hypertrophic obstructive cardiomyopathy, by directly modulating sarcomeric function. These agents may move earlier in the treatment pathway for selected patients with HOCM, although longer-term data, access, monitoring burden and cost-effectiveness remain important considerations.

## Background

Hypertrophic cardiomyopathy (HCM) is defined as increased left ventricular (LV) wall thickness or mass of more than 15 mm that cannot be attributed to loading conditions, including hypertension and alular disease. In children, the absolute LV wall thickness is related to body surface area and must exceed 2 standard deviations above the predicted mean to meet criteria for HCM (1, 2).

In HCM, the proportion of myosin heads in an “off state”, where they use minimal energy, is disrupted. Typically, in a healthy heart, 40%–50% of myosin heads should be “off”; however, in HCM this is reduced by more than half, with only 15%–20% of the myosin heads remaining in this “off” state. The additional myosin heads in the “on” state consume more energy in the form of adenosine triphosphate (ATP) and are more likely to interact with actin, leading to an increase in myosin–actin cross-bridges during both systole and diastole, resulting in inefficient, overly forceful contraction and impaired relaxation of the heart. Persistent sarcomeric hyperactivity triggers pro-hypertrophic, pro-inflammatory, and pro-fibrotic pathways, ultimately leading to progressive structural changes in the heart, including fibrosis, disorganised myofilaments or myocyte disarray, and increased mechanical stress (3–6).

HCM is classed as hypertrophic obstructive cardiomyopathy (HOCM) when there is left ventricular outflow tract obstruction (LVOTO) caused by systolic narrowing of the outflow tract in combination with functional changes in the heart; this will be the main focus of this review. LVOTO occurs because of hypertrophy of the interventricular septum and its interaction with the mitral valve and its systolic anterior motion (SAM) (6, 7). The combined processes of LVOTO are associated with dangerous ventricular arrhythmias due to increased myocardial stress (8, 9).

HCM is typically inherited in an autosomal dominant manner, caused by variants in sarcomere protein genes that encode components of the heart's contractile machinery (10). Rarely, both copies of the gene will be defective, a condition that may lead to a more severe manifestation of the disease (11). The most common gene variants implicated in HCM include MYH7, MYBPC3, TNNT2, and TPM1, encoding the β-myosin heavy chain, cardiac myosin-binding protein C, cardiac troponin T and *α*-tropomyosin respectively, and accounting for 30%–40% of HCM cases (12–14). The prevalence of HCM in the general population is historically estimated to be approximately 1 in 500 individuals and it is the most common identifiable cause of sudden cardiac death in young people under 35 (15, 15). However, newer estimates indicate prevalence could be as high as 1:300 or 1:200 (16, 17). Despite this, the overall survival of most patients with HCM is comparable to an age- and sex-matched population without heart disease (18, 19). Many individuals with HCM are either asymptomatic or experience only mild symptoms. Additionally, many carriers of HCM-associated genetic variants may not develop clinically detectable disease, with reported penetrance 57% (20).

Symptoms of HOCM include dyspnoea, angina, palpitations, syncope, fatigue and sudden cardiac death (15, 21). Implantable cardioverter defibrillator (ICD) implantation may be considered as primary and secondary prevention in HOCM patients. The ESC emphasises a need for a comprehensive risk assessment approach, considering multiple factors to determine the necessity of an ICD (1). The HCM Risk-SCD calculator is used in clinical practice. Important risk factors considered for ICD Implantation (22):

1. Age
2. LVOT gradient
3. Left atrial diameter
4. Family history of SCD: A history of SCD in first-degree relatives with HCM or any relative under 40 years of age.
5. Unexplained syncope: Episodes of fainting without a clear cause, suggesting arrhythmic events.
6. Nonsustained ventricular tachycardia (NSVT): Detected through ambulatory (Holter) monitoring.
7. Maximal left ventricular wall thickness: Significant hypertrophy, typically ≥30 mm.

An ICD is not indicated with a 5-year sudden cardiac death (SCD) risk of less than 4%, whilst ICD implantation is recommended in patients with HCM-SCD risk above 6%. ICD may be considered in HOCM patients with risk between 4%–6%, based on additional factors (23–29). It is important to consider cumulative risk burden, patient preferences, and overall clinical context, and shared decision making is pivotal (30). Along with the ESC HCM Risk-SCD model, the ACC/AHA risk-stratification framework helps guide SCD risk stratification internationally and both are well incorporated into clinical practice (31).

The objective of this review article is to provide a comprehensive overview and breakdown of current available treatment options, including traditional medical therapies and surgical septal reduction therapies in the era of new emerging pharmacologic options, including myosin inhibitors, and suggestions of where the disease treatment may progress in the coming years.

## Lifestyle modifications

HOCM was first formally categorised in the mid 20th century and patients with the condition were traditionally recommended to avoid strenuous activity, due the increased risk of sudden cardiac death, and optimised hydration due to the resultant increase in left ventricular outflow tract (LVOT) gradients associated with dehydration and the related decrease in cardiac preload (32, 33). The 2023 European guidelines were more specific, encouraging regular low- to moderate exercise in all individuals with cardiomyopathy (Class 1C recommendation), and advocating the consideration of high-intensity exercise and even competitive sport in asymptomatic and low-risk individuals with HCM in the absence of inducible LVOTO or arrhythmias (Class IIb recommendation) (1).

## HOCM therapies

### Medications

For symptomatic relief, non-vasodilating beta blockers, titrated up to the maximum tolerated dose, have been recommended in HOCM treatment since the mid 20th century, reducing LVOTO via a reduction in heart rate and heart contractility leading to improved diastolic filling (1, 34). There is no definite evidence or guidance as to which beta-blocker should be used preferentially, however Metoprolol has been shown to significantly reduce LVOT gradients before, during, and after exercise (reduced by 47mmHg, 34mmHg, and 70mmHg respectively), and improved both symptoms and quality of life in HOCM patients in one of the few randomised controlled trials in HCM prior to the current myosin inhibition era (35). In the BASIC study, Bisoprolol treatment led to a mean LVOT gradient reduction of 28mmHg. 36% of patients had a reduction to less than 30 mmHg, 62% of patients to less than 50 mmHg, and 33% of patients experienced an improvement of at least one New York Heart Association (NYHA) class (36). At present, there are a lack of comparative studies between beta-blockers and their therapeutic effect in HCM, therefore considerations should include the medicine's efficacy for the specific indication, along with its tolerability and specific characteristics including cardioselectivity (37). Relative contraindications to beta-blockers include asthma, COPD, bradycardia, decompensated heart failure and severe peripheral artery disease (38). Beta-blocker side effects include hypotension, dizziness and bradycardia (39).

Verapamil, a phenylalkylamine non-dihydropyridine calcium channel blocker (CCB), has played a role for decades in symptomatic relief in HOCM (40–43). Verapamil blocks the entry of calcium ions through L-type calcium channels in cardiac and vascular smooth muscle cells during depolarisation, leading to relaxation of coronary vascular smooth muscle, promoting coronary vasodilation, and enhancing myocardial oxygen supply. Additionally, Verapamil has an effect by reducing heart rate and modulating sympathetic nervous system activity, although it may be associated with adverse chronotropic effects (1, 33, 44). ESC recommended starting Verapamil dose is 40 mg three times daily, increasing up to a maximum of 480 mg daily (1). Verapamil is contraindicated in conjunction with beta blockers due to the risk severe bradycardia, hypotension and conduction block (45). Furthermore, it is advised not to use Verapamil in severe obstruction or cases with raised pulmonary pressure due to the risk of pulmonary oedema (1). Common side effects of Verapamil include headaches, palpitations, dizziness, constipation and ankle oedema (46).

Diltiazem, another calcium-channel blocker used in HOCM, is a benzothiazepine non-dihydropyridine CCB and blocks L-type calcium channels in a less selective manner than Verapamil. Diltiazem has class 1 indication for HOCM, often used where Verapamil is not well tolerated as its biochemistry makes it less cardioselective (1, 47–52). Recommended starting dose of Diltiazem is 60 mg three times daily, up to a maximum of 360 mg (1). Diltiazem has a similar side effect profile to Verapamil although sometimes more severe, with oedema and headache more common (53–55).

Disopyramide, a class IA antiarrhythmic agent, functions as a sodium channel blocker with strong negative inotropic effects and is particularly effective in reducing LVOT gradients in adults with HOCM. Disopyramide is typically prescribed for patients with HOCM who continue to experience symptoms despite treatment with beta-blockers or calcium-channel blockers and exhibit evidence of LVOT obstruction, as additional therapy rather than as first-line monotherapy. It is usually used in combination with an atrioventricular nodal blocking agent, such as a beta-blocker or non-dihydropyridine calcium-channel blocker, to reduce the risk of enhanced atrioventricular conduction and tachyarrhythmia (56, 57, 161). Disopyramide is associated with several side effects including constipation, urinary retention, blurred vision, dry mouth, hypotension and dizziness, primarily due to its anticholinergic properties (58). Furthermore, routine QTc monitoring is necessary with disopyramide use and the dose must be reduced if the QTc exceeds 500 ms. Disopyramide is contraindicated in patients already taking other medicines which prolong the QTc, as well as patients with glaucoma and prostatism. Caution is also required in patients with significant conduction disease or a history of ventricular arrhythmia. Importantly, patients taking Disopyramide may experience the phenomenon of tachyphylaxis, with early symptomatic improvement (>80%) not maintained in 33% of the 51 children with HOCM in a 2023 study, the largest study to date investigating the use of disopyramide in childhood HOCM (58). This loss of response is clinically relevant because it may limit the long-term effectiveness of Disopyramide as a strategy to defer escalation to septal reduction therapy or myosin inhibition. There have been considerable supply chain issues sourcing disopyramide in the United Kingdom in recent years, and lack of availability has impacted on patient treatment and symptom burden. Despite these limitations, Disopyramide remains an important component of guideline-directed pharmacological therapy for symptomatic obstructive HCM when symptoms persist despite beta-blocker or calcium-channel blocker therapy.

## Surgical myectomy and alcohol septal ablation (ASA)

### Surgical procedures

Ventricular septal myectomy is the primary surgical treatment for LVOTO. It is an open-heart surgical procedure carried out under general anaesthesia and involves removing hypertrophied tissue from the basal septum to reduce LVOTO. A portion of the basal septum is excised to create a rectangular trough that extends distally, beyond the area where the mitral leaflet contacts the septum and lies below the aortic valve. The procedure begins with a median sternotomy to access the heart; the heart is temporarily stopped, and a heart-lung machine takes over the role of circulating blood and oxygenating the body. Access to the LVOT and interventricular septal muscle is achieved through an aortotomy. Intraoperative transoesophageal echocardiography (TOE) guides the precise identification of the muscle to be resected (Figure 1). The resection is performed incrementally to avoid over-resection, which can result in complications such as mitral valve damage or injury to the conduction system, including the bundle of His and the atrioventricular (AV) node, both of which are located near the interventricular septum (59). A permanent pacemaker may be needed (≈5.6%) in cases of significant conduction disturbances postoperatively e.g., complete atrioventricular block (60–62).

**Figure 1:**
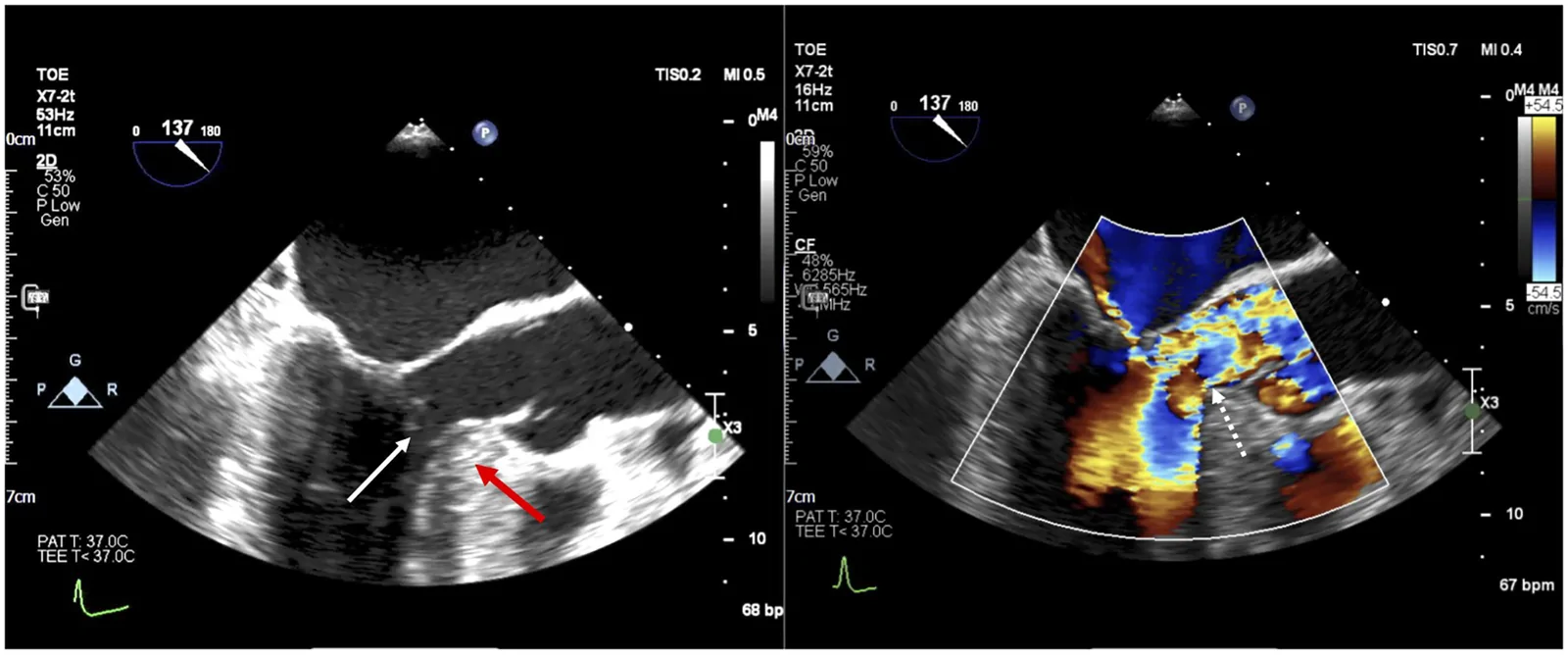
Transoesophageal echocardiogram in a patient with hypertrophic obstructive cardiomyopathy. Left panel—2-dimensional imaging showing systolic anterior motion of the mitral valve (white arrow) and basal septal hypertrophy (red arrow); causing severe left ventricular outflow tract obstruction manifesting as flow acceleration seen in the right panel, highlighted in the corresponding colour flow Doppler comparative image (dotted arrow).

Surgical myectomy is often accompanied by additional procedures to address associated cardiac anomalies in around 30% of cases, with 9% undergoing mitral valve repair, which is indicated when there is associated severe mitral regurgitation or pronounced SAM with LVOTO (63, 64). Additionally, addressing anomalies of the papillary muscles, coronary artery bypass grafting (CABG) (≈5%), Cox-Maze IV procedure for addressing atrial fibrillation and excision of anomalous muscle bundles are examples of additional available therapeutic options when undergoing surgical myectomy (65–67).

Myectomy effectively reduces LV outflow tract gradients in more than 90% of patients, minimises mitral regurgitation caused by SAM and significantly enhances exercise tolerance and symptom relief (1, 59). While combining CABG with surgical myectomy can address two conditions simultaneously, there is a risk of increased mortality and adverse effects in patients undergoing combined procedures compared to those undergoing isolated septal myectomy (68). The risk of concomitant CABG at the time of ventricular septal myectomy is reduced when performed at experienced centres (69). In patients requiring mitral valve intervention, MV repair is preferred to MV replacement as a concomitant procedure with myectomy due to better outcomes (70).

### Alcohol septal ablation

Alcohol septal ablation (ASA) is a minimally invasive alternative to surgical myectomy, performed in experienced centres for patients with HOCM. The procedure, performed percutaneously, involves selectively injecting 1–3 mL absolute ethanol (96%–100% concentration) into the first septal perforator branch of the left anterior descending (LAD) coronary artery, creating a scar to reduce LVOTO by causing localised myocardial necrosis. A balloon catheter is positioned in the selected septal artery, through which absolute ethanol is injected; the balloon occludes the vessel and prevents ethanol entering other branches or the left anterior descending coronary artery. Alcohol induces a controlled myocardial infarction in the targeted tissue, leading to septal thinning, LVOT widening, and gradient reduction as the necrotic myocardium is replaced by scar tissue over time (71, 72). ASA is particularly effective in individuals with: 1. septal thicknesses of ≥17 mm, as this ensures there is sufficiently hypertrophied myocardium to target during the procedure; and 2. coronary anatomy that allows precise targeting of septal arteries (72).

ASA is especially prevalent in Europe, where it is performed more frequently than in other regions (73, 74). Clinical outcomes, including gradient reduction, symptom alleviation, and improved exercise tolerance, are similar to those achieved with surgical myectomy (75, 76). ASA significantly reduces symptoms such as dyspnoea, chest pain, and syncope while improving exercise capacity. Approximately 90% of patients experience an improvement in functional status, reducing to NYHA class I or II after the procedure (72). Potential complications include conduction disturbances, such as heart block requiring pacemaker implantation (5%–15%), ventricular arrhythmias (1%–2%), and coronary artery damage (77).

## Comparative indications and outcomes for alcohol septal ablation (ASA) and surgical myectomy

Guidelines align closely amongst major cardiovascular societies, emphasising the importance of symptom relief and gradient reduction in patients with significant LVOTO (33, 78, 79).

ASA and surgical myectomy have comparable clinical and haemodynamic indications, including symptoms (NYHA class II/III), hypertrophic obstructive cardiomyopathy (HOCM) with LVOT gradient of ≥50 mmHg, and a lack of response to medical therapy. However, the choice of treatment depends on patient-specific factors, such as demographics, anatomical suitability, site-specific expertise, and procedural risks.

ASA remains an important alternative to surgery, particularly for older or higher-risk patients, as it is a minimally invasive procedure that avoids the need for general anaesthesia or cardiopulmonary bypass. However, it is less effective in cases of complex septal anatomy, anomalous coronary arteries, or where associated mitral valve or subvalvular abnormalities require direct correction (59, 70, 80–85). Surgical myectomy is often the treatment of choice for younger or lower surgical risk patients, those with substantial septal hypertrophy (>30 mm), or individuals with additional structural abnormalities, such as mitral valve dysfunction, that can be corrected during surgery. These key differences are summarised in Table 1 (70, 72).

**Table 1:** Comparison of surgical septal myectomy and alcohol septal ablation in obstructive HCM.

Feature	Surgical septal myectomy	Alcohol septal ablation
**Procedure type**	Open surgical septal reduction performed under general anaesthesia with cardiopulmonary bypass.	Percutaneous catheter-based septal reduction using targeted ethanol injection into a septal perforator artery.
**Typical patient selection**	Often preferred in younger patients, those at low surgical risk, patients with marked septal hypertrophy, or those with associated mitral, subvalvular or other structural abnormalities requiring correction.	Often considered in older patients, patients at higher surgical risk, or those who are unsuitable for surgery but have favourable septal perforator anatomy.
**Septal thickness/anatomical considerations**	Particularly useful in severe septal hypertrophy, including marked hypertrophy >30 mm, and in complex LVOT or mitral/subvalvular anatomy.	Requires suitable septal perforator anatomy and sufficient target septal thickness, commonly ≥17 mm, to allow controlled infarction of the hypertrophied basal septum.
**Effect on LVOT gradient**	Provides immediate and usually substantial LVOT gradient reduction, often confirmed intraoperatively by echocardiography.	Can produce substantial LVOT gradient reduction, but the full effect may evolve gradually over weeks to months as septal thinning and remodelling occur.
**Durability**	Generally the most durable septal reduction strategy, with low rates of recurrent obstruction or repeat intervention in experienced centres.	Effective in selected patients, but durability may be less predictable, with some patients requiring repeat ablation or subsequent myectomy.
**Pacemaker risk**	Risk of complete heart block and permanent pacemaker implantation is present but generally lower than with ASA in experienced centres.	Higher risk of conduction disturbance and permanent pacemaker implantation, particularly because of alcohol-induced septal infarction close to the conduction system.
**Ability to address mitral valve or subvalvular abnormalities**	Allows direct correction of associated mitral valve disease, systolic anterior motion, anomalous papillary muscles, muscle bundles or other structural contributors to LVOTO.	Does not allow direct surgical correction of mitral valve, papillary muscle or subvalvular abnormalities.
**Recovery profile**	Requires sternotomy or surgical access, inpatient recovery and post-operative rehabilitation.	Less invasive, usually with shorter procedural recovery, although haemodynamic improvement may be delayed.
**Main limitations**	Requires specialist surgical expertise and carries risks associated with open cardiac surgery. Outcomes are strongly influenced by centre and operator experience.	Dependent on suitable coronary anatomy; risk of unintended infarction, conduction disease, residual obstruction and need for reintervention.
**Overall role**	Preferred definitive therapy when durable relief is required, anatomy is complex, or concomitant surgical correction is needed.	Important alternative for selected patients, especially those unsuitable for surgery and with favourable anatomy.

Surgical myectomy is generally a safe procedure (86, 87). A 2005 study of 289 patients who underwent myectomy reported survival rates at 1, 5 and 10 years of 98%, 96% and 83%, respectively, comparable to the age- and sex-matched general population (88). Furthermore, for children, a study which followed patient outcomes of a population of 1999 over 45+ years showed operative mortality at 1%, and the cumulative incidence of redo myectomy was 5.8% at 5 years and 8.3% at 10 years, indicating favourable long-term results in younger patients (89).

While both surgical myectomy and ASA can lead to significant symptomatic improvement, surgical myectomy may offer advantages in terms of greater LVOT gradient reduction, lower reintervention rates and reduced pacemaker risk (75, 76).

An important point is the experience of the operator and the centre in which the procedures are carried out. A 2023 study investigating 19,007 recipients of SRT between 2010 and 2019 showed that surgical myectomy outcomes were significantly influenced by hospital volume, while alcohol septal ablation outcomes did not show the same dependency (90, 91). However, operator experience remains important for both procedures, with increased 30-day mortality reported when procedures are carried out by low-volume operators (91).

## Percutaneous intramyocardial septal radiofrequency ablation (PIMSRA)

An alternative to surgical options is percutaneous intramyocardial septal radiofrequency ablation data (PIMSRA). The procedure is performed through a small incision, a radiofrequency electrode is inserted into the interventricular septum allowing for radiofrequency energy to be delivered, in a controlled manner, to alleviate LVOTO. There are less available data for PIMSRA, and it is a relatively new procedure, with initial research published in 2018 (92, 93); it may be a suitable non-surgical alternative to be explored in the future (94).

## Myosin inhibitors: A new era in pharmacologic management

### Class mechanism and monitoring considerations

Myosin inhibitors, such as Mavacamten (first-in-class) and Aficamten (FDA and NICE approved), offer a targeted approach to addressing the hypercontractility characteristic of hypertrophic obstructive cardiomyopathy (HOCM), by directly modulating sarcomeric function. Mavacamten, approved by the FDA in 2022 and the MHRA and NICE in 2023, represents a novel and first-in-class therapeutic option by stabilizing myosin heads in their inactive state bound to ATP, preventing their interaction with actin and limiting cross-bridge cycling. The therapeutic mechanism of myosin inhibition reduces myocardial contractility (95–98). As a therapeutic class, cardiac myosin inhibitors require careful dose titration and echocardiographic monitoring, particularly of left ventricular ejection fraction (LVEF), to avoid excessive suppression of systolic function. Dose adjustment or temporary interruption may be required if clinically significant LVEF reduction occurs.

By alleviating the forces that displace the mitral valve into the LVOT, myosin inhibitors can modulate systolic anterior motion (SAM) of the mitral valve and reduce associated mitral regurgitation, in a similar way to invasive SRT (99, 100). Beyond their immediate haemodynamic benefits, these agents lower myocardial energy demand, which may help mitigate long-term stress, maladaptive remodelling, and fibrosis (101–104).

### Mavacamten: evidence and clinical application

PIONEER-HCM was a 12-week, non-randomised phase 2 trial with reduction in post-exercise LVOT up to 89mmHg for one of the groups. Resting LVOT gradients along with exercise capacity were also shown to improve (105). Mavacamten was shown to reduce LVEF in a concentration-dependent manner, and the side effect profile was relatively unremarkable (106).

The success of the PIONEER trial led to the EXPLORER-HCM trial, which was a pivotal Phase 3 randomised, double-blind, placebo-controlled study designed to evaluate the efficacy and safety of Mavacamten in patients, confirming significant improvement in symptoms, enhanced exercise capacity, and effective reduction of LVOTO in patients with HOCM (95, 96, 107, 108, 110, 111, 112). Further exploration revealed improvements in electrocardiogram (ECG) morphology and NT-proBNP levels (113).

EXPLORER-HCM enrolled 251 adult patients with NYHA class II or III symptoms and an LVOT gradient of ≥50 mmHg at rest or during provocation, despite stable background therapy with beta-blockers or calcium-channel blockers. Participants were randomised in a 1:1 ratio to receive either Mavacamten or placebo for 30 weeks. The primary endpoint assessed composite functional improvement, with secondary endpoints including reductions in LVOT gradient, improvements in NYHA class, and quality-of-life metrics assessed using the Kansas City Cardiomyopathy Questionnaire (KCCQ) and other tools (112, 114, 115).

The EXPLORER-HCM results demonstrated a significant benefit with Mavacamten treatment, with 37% of patients in the Mavacamten group achieving the primary endpoint, compared to 17% in the placebo group (*p* < 0.001). Mavacamten significantly reduced resting LVOT gradients, improved NYHA class and quality-of-life scores, and was generally well tolerated. However, a small number of patients experienced reversible reductions in left ventricular ejection fraction, emphasising the need for close monitoring. These findings established Mavacamten as an effective and targeted treatment for HOCM, significantly improving functional capacity, reducing symptoms, and lowering LVOT gradients (105, 112, 116, 117). Subsequent studies have shown similar findings with up to 80% having LVOTO eliminated after 6 months of the drug (118). Sustained clinical and haemodynamic benefits have been reported through to 180 weeks (119). These long-term extension data are important because they suggest that the benefits of Mavacamten can be maintained with continued therapy, although ongoing LVEF surveillance remains necessary.

Beyond obstructive HCM, research is exploring the application of myosin inhibitors in non-obstructive HCM (63). MAVERICK-HCM investigated Mavacamten's potential use as a therapy in non-obstructive HCM with significant reductions in NT-proBNP and cTnI (116). However, no statistically significant improvement in exercise capacity or patient-reported health status was demonstrated with Mavacamten in non-obstructive HCM over placebo in ODYSSEY-HCM, a 580-person, phase 3, international, randomised, double-blind, placebo-controlled trial focusing on adults with symptomatic non-obstructive HCM (NYHA class II–III) over 48 weeks (120). These results suggest distinct pathophysiology in non-obstructive HCM compared with obstructive disease, perhaps limiting clinical benefits of Mavacamten to HOCM (121).

### Aficamten: evidence and emerging role

Aficamten is a second-in-class myosin inhibitor. Its clinical evidence base is newer than that of Mavacamten, but phase 2 and phase 3 data suggest an important emerging role in obstructive HCM. The SEQUOIA-HCM trial was a phase 3, double-blind, randomised, placebo-controlled study designed to assess the efficacy and safety of Aficamten (122). SEQUOIA-HCM showed improvement in patients' exercise capacity, symptoms, LVOT gradient and mitral regurgitation, while being well tolerated with serious adverse events in only 5.6% of the 282 participant study (122, 123).

Aficamten dose titration is necessary to optimise efficacy while avoiding excessive suppression of myocardial contractility. Patients require regular monitoring of left ventricular ejection fraction (LVEF) during treatment to ensure safety and effectiveness (103, 121, 124). This mirrors the broader class requirement for LVEF surveillance, as the therapeutic effect depends on reducing hypercontractility without causing clinically significant systolic dysfunction.

The REDWOOD-HCM trial highlighted the potential of Aficamten in enhancing exercise capacity, alleviating symptoms of heart failure and improving cardiac biomarkers in non-obstructive HCM (121). More recently, the MAPLE-HCM study demonstrated superiority of Aficamten over metoprolol in obstructive HCM (125). Consequently, there is potential for myosin inhibitors to move earlier in the treatment pathway for selected patients with HOCM, although longer-term data, guideline updates, cost-effectiveness analyses and real-world implementation studies are needed before they can be considered replacements for established first-line therapy. Further to the neutral results of the ODYSSEY-HCM study of Mavacamten, the role of myosin inhibition in non-obstructive HCM was reassessed in the ACACIA-HCM trial, evaluating Aficamten's use for this clinical indication. The study showed Aficamten safely and effectively improved exercise capacity and daily symptoms for patients with non-obstructive hypertrophic cardiomyopathy, meeting its primary goals (126). However, it should be noted the treatment effect, as evidenced by improvement in peak oxygen uptake during cardiopulmonary exercise testing was less substantial in non-obstructive HCM patients in ACACIA-HCM compared to the equivalent Aficamten trial in HOCM, SEQUOIA-HCM (mean change 0.64 mL/kg/min vs. 1.8 mL/kg/min).

### Clinical positioning and unresolved questions

A systematic review and meta-analysis further corroborated the positive study findings in HOCM patients, indicating that cardiac myosin inhibitors effectively improve NYHA class and quality of life (127, 128). The European Society of Cardiology (ESC) has acknowledged the role of myosin inhibitors in their guidelines, recommending their consideration for patients with HCM and LVOTO who remain symptomatic despite optimal medical therapy (1). As the therapeutic landscape evolves, ongoing studies continue to refine the role of myosin inhibitors, potentially extending their use to a broader spectrum of cardiomyopathies and contributing to a more nuanced approach in cardiovascular care. Guidelines specifically mention Mavacamten, but future guidance is likely to be broadened to myosin inhibitors as a drug class.

At present, Mavacamten has the more mature evidence base, and emerging long-term extension data, whereas Aficamten has rapidly developing phase 3 evidence and will offer an important second-in-class option. Important unresolved questions of myosin inhibition include long-term durability, cost-effectiveness, access, monitoring burden, and the uncertain role of the drug class in non-obstructive HCM. The key clinical studies informing the current evidence base for Mavacamten and Aficamten are summarised in Table 2.

**Table 2:** Key clinical studies of cardiac myosin inhibitors in hypertrophic cardiomyopathy.

Study	Drug	Population	Design	Key findings	Clinical relevance
PIONEER-HCM	Mavacamten	Symptomatic obstructive HCM	Phase 2, non-randomised, 12-week study	Reduced post-exercise LVOT gradients, with improvements in symptoms and exercise capacity.	Provided early proof-of-concept that direct myosin inhibition could reduce obstruction and improve functional status in obstructive HCM.
EXPLORER-HCM	Mavacamten	Adults with symptomatic obstructive HCM, NYHA class II–III, LVOT gradient ≥50 mmHg	Phase 3, randomised, double-blind, placebo-controlled trial	37% of patients receiving Mavacamten met the primary composite endpoint compared with 17% receiving placebo. mavacamten reduced LVOT gradients, improved NYHA class, exercise capacity and quality-of-life measures.	Established Mavacamten as an effective disease-specific pharmacologic therapy for symptomatic obstructive HCM.
MAVA-LTE/long-term extension data	Mavacamten	Patients with obstructive HCM continuing Mavacamten after initial trial participation	Long-term extension follow-up	Sustained clinical and haemodynamic benefits have been reported through long-term follow-up, with ongoing need for LVEF surveillance.	Supports durability of benefit during continued treatment, while highlighting the importance of long-term monitoring.
VALOR-HCM	Mavacamten	Patients with obstructive HCM referred for septal reduction therapy despite maximally tolerated medical therapy	Phase 3, randomised, double-blind, placebo-controlled trial	At 16 weeks, only 17.9% of Mavacamten-treated patients remained eligible for septal reduction therapy, compared with 76.8% in the placebo group.	Demonstrated that Mavacamten can substantially reduce short-term eligibility for invasive septal reduction therapy in selected patients.
MAVERICK-HCM	Mavacamten	Symptomatic non-obstructive HCM	Phase 2 randomised, double-blind, placebo-controlled trial	Reduced biomarkers including NT-proBNP and cardiac troponin I, but clinical efficacy in non-obstructive HCM remained uncertain.	Suggested biological activity in non-obstructive HCM but did not establish clear symptomatic or functional benefit.
ODYSSEY-HCM	Mavacamten	Adults with symptomatic non-obstructive HCM, NYHA class II–III	Phase 3, international, randomised, double-blind, placebo-controlled trial	Did not demonstrate statistically significant improvement in exercise capacity or patient-reported health status compared with placebo.	Indicates that benefits of Mavacamten may not translate directly from obstructive to non-obstructive HCM, supporting separate evaluation of these disease phenotypes.
REDWOOD-HCM	Aficamten	Obstructive HCM, with additional exploration in non-obstructive HCM cohorts	Phase 2 multicentre, randomised, double-blind, placebo-controlled trial	Demonstrated reductions in LVOT gradients and improvements in symptoms and cardiac biomarkers, with dose titration guided by echocardiographic monitoring.	Provided early evidence supporting Aficamten as a next-generation cardiac myosin inhibitor.
SEQUOIA-HCM	Aficamten	Symptomatic obstructive HCM	Phase 3, randomised, double-blind, placebo-controlled trial	Improved exercise capacity, symptoms, LVOT gradients and mitral regurgitation, with serious adverse events reported in a minority of patients. Among patients eligible for septal reduction therapy at baseline, 88% treated with Aficamten were no longer eligible after 24 weeks.	Supports Aficamten as an effective second-in-class myosin inhibitor and further strengthens the role of myosin inhibition as an alternative to immediate septal reduction therapy.
MAPLE-HCM	Aficamten versus Metoprolol	Symptomatic obstructive HCM	International, double-blind, double-dummy, comparative clinical trial	Demonstrated superiority of Aficamten over Metoprolol in obstructive HCM with significantly greater improvement in peak oxygen uptake at 24 weeks with supportive findings across secondary endpoints.	Raises the possibility that myosin inhibitors may move earlier in the treatment pathway and potentially challenge beta-blockers as default first-line therapy in selected patients.
ACACIA-HCM	Aficamten	Symptomatic non-obstructive HCM	Phase 3, randomised, double-blind, placebo-controlled trial	Aficamten was shown to result in significantly greater change in exercise capacity vs placebo in non-obstructive HCM	Met the primary end-points, unlike the equivalent Mavacamten trial in non-obstructive HCM, ODYSSEY-HCM. However, the improvement in exercise capacity in both studies was substantially less than the HOCM trials. More studies are needed in this patient group

## Discussion: invasive septal reduction therapies and myosin inhibitors

The onset of action of myosin inhibitors is gradual, due to their mechanism of action, typically requiring 4–8 weeks to achieve significant reductions in LVOT gradients, although improvements can be seen as early as two weeks (110, 111, 129). Similarly, alcohol septal ablation has a delayed onset of action; the procedure leads to gradual thinning and remodelling over several weeks to months after iatrogenic myocardial necrosis; while initial LVOT gradient reductions may be observed within minutes, the full therapeutic effect often takes 3–12 months to become apparent (83, 130). In contrast, surgical septal myectomy provides immediate relief of LVOTO, as the hypertrophied septal tissue is directly removed during the procedure. Intraoperative echocardiography confirms the reduction in LVOT gradient at the time of surgery, making the effect instantaneous. However, the recovery from open heart surgery is significant, typically 2–3 months and requiring rehabilitation, along with other post-surgical risks (131).

Myosin inhibitors effectively reduce LVOT gradients, typically achieving reductions of 30–50 mmHg. Although these reductions are substantial, residual gradients may persist in some patients (109, 132). Conversely, surgical septal myectomy more consistently achieves robust and near-complete gradient reductions to levels below 10–20 mmHg (133, 134). ASA also reduces LVOT gradients significantly, but its efficacy is less predictable compared to surgical myectomy. Residual gradients may remain in some cases, especially when septal artery anatomy limits the extent of the inducible infarction (135).

The sustainability of outcomes with myosin inhibitors depends on long-term adherence to therapy, however without the risks of invasive treatments. Discontinuation of the medication often results in the recurrence of LVOTO and symptoms (136). While these agents show promise for preventing disease progression, the true extent of long-term benefits is still under investigation. In contrast, surgical septal myectomy provides durable results, with most patients experiencing lasting relief of obstruction and symptoms. Recurrence of LVOTO is rare but may occur in fewer than 5% of cases due to progressive disease or an inadequate initial resection. Alcohol septal ablation offers less durable outcomes than surgical myectomy. Recurrence of obstruction may occur due to incomplete septal thinning or disease progression, with approximately 10%–15% of patients requiring repeat procedures (137, 138).

The VALOR-HCM assessed patients who had been referred for SRT on maximally tolerated medication, including Disopyramide, to ascertain whether Mavacamten can be used in the context of SRT-eligible patients. VALOR-HCM reported only 17.9% of Mavacamten patients remained SRT-eligible at 16 weeks vs. 78% in the placebo group (101). This phenomenon was also seen in the SEQUOIA-HCM trial with Aficamten; among patients who were eligible for SRT at baseline, 88% of those treated with Aficamten were no longer eligible for SRT after 24 weeks (122). Given the risks of SRT, the data supporting the role of myosin inhibitors to delay the need for SRT, if not remove it as an option, is promising (139).

Myosin inhibitors provide a pharmacological alternative to septal reduction therapies, offering a non-invasive treatment with a gradual onset of action and significant, though slightly less robust, LVOT gradient reduction. VALOR-HCM and SEQUOIA-HCM suggest many SRT-eligible patients may become ineligible after treatment. Medical therapy makes myosin inhibition particularly appealing for patients who are not candidates for invasive procedures. Surgical myectomy remains the gold standard for durable and immediate relief of LVOTO in patients with severe hypertrophy or complex anatomy but involves major surgery requiring a prolonged period of recovery. Alcohol septal ablation, while less invasive, offers intermediate outcomes and is best suited for patients unable to undergo surgery who have favourable septal artery anatomy. The choice of therapy should be individualised based on patient-specific factors, including anatomy, comorbidities and personal preferences, with involvement of the multidisciplinary team (MDT) (85, 105, 112, 140–143). A summary of the main treatment options, indications, benefits, limitations and guideline recommendations is provided in Table 3.

**Table 3:** Treatment summary table.

Therapy	Type for HOCM	Indication	Benefits	Predictability of effect	Key risks/limitations	ESC (2023) Class/LOE	AHA/ACC (2024) Class/LOE
Beta-blockers (non-vasodilating)	Medical	First-line for symptomatic resting or provocable LVOTO; Mild symptoms (NYHA I–II)	Symptom relief; reduces gradients in many; widely available	Inconsistent	Fatigue, bradycardia, hypotension; may limit exercise tolerance	I/B	I/B-NR
Non-dihydropyridine CCBs (verapamil/diltiazem)	Medical	Alternative if beta-blockers ineffective/not tolerated (symptomatic LVOTO); Mild symptoms (NYHA I–II)	Symptom relief (selected patients)	Inconsistent	Hypotension, constipation, bradycardia; verapamil potentially harmful with very high gradients/severe dyspnoea at rest	I/B	I/B-NR (verapamil); I/C-LD (diltiazem)
Disopyramide (with AV-nodal blocker)	Medical	Add-on for persistent symptoms despite BB/CCB (symptomatic LVOTO)	Negative inotrope; can reduce LVOT gradient and improve symptoms; may help defer escalation to septal reduction therapy in selected patients	Variable; initial response may diminish over time	Anticholinergic effects (including dry mouth, constipation and urinary retention); QT prolongation requiring baseline and follow-up ECG monitoring; avoid or use cautiously with other QT-prolonging drugs, glaucoma, prostatism or significant conduction disease; tachyphylaxis may limit long-term effectiveness	I/B (add-on)	I/B-R
Myosin inhibitors (class; e.g., Mavacamten)	Medical	Second line; Persistent symptoms despite BB/CCB (adult symptomatic LVOTO) as escalation; Moderate to severe symptoms (NYHA III–IV),	Reduces obstruction and improves symptoms/exercise capacity in responders	Consistent	LVEF reduction risk → echo surveillance; drug interactions; cost/access; long-term data accruing (agent-specific)	IIa/A (Mavacamten add-on)	I/B-R (myosin inhibitor)
Alcohol septal ablation (ASA)	Procedural (catheter-based)	Severe symptoms refractory to medical therapy; typically when surgery contraindicated/high risk and suitable anatomy	Minimally invasive; reduces gradient; shorter recovery vs surgery	Variable	Procedural risk: AV block/pacemaker, coronary complications, infarct control, residual obstruction/re-intervention	I/B (SRT); IIa/C (catheter-based in selected)	I/C-LD (when surgery contraindicated/high risk)
Surgical septal myectomy	Surgical	Severe symptoms refractory to medical therapy; anatomy favours surgery or concomitant cardiac surgery/mitral issues	Durable gradient + symptom relief; can address mitral/subvalvular abnormalities	Highly reliable	Surgical risks; requires experienced HCM centre	I/B (SRT); IIa/C (selected features/concomitant surgery)	I/B-NR (SRT); I/B-NR (with associated surgical disease)

Structural Heart MDT typically includes cardiologists, cardiac surgeons, imaging specialists, and interventional cardiologists, who collectively assess the patient's clinical profile, LVOT gradient severity, anatomical features, and comorbidities. A collaborative decision-making process helps identify the most appropriate treatment pathway, balancing efficacy, risks, and patient preferences. By leveraging the expertise of various specialists, the MDT ensures that complex cases are managed optimally.

## Future directions and evolving role of each treatment

### Gene therapy

There is an emerging role for gene therapy in the treatment of genetic diseases. Gene therapy, targeting and replacing/editing defective genes, has been shown to provide cures for previously debilitating or terminal genetic makeups (144–147). In relation to HCM, AAV9-mediated MYBPC3 gene therapy has shown promising symptomatic improvements in mice models (148, 149). Also, a landmark first-in-human study, *in vivo* CRISPR–Cas9 gene editing using lipid nanoparticle delivery achieved large, dose-dependent and durable reductions in circulating transthyretin following a single intravenous infusion in patients with ATTR amyloidosis, establishing the feasibility of permanent, mechanism-based gene targeted gene editing in humans (150). This work provides a compelling exemplar of how gene-based therapies could enable durable disease modification beyond chronic pharmacological treatment in HOCM. However, many hurdles remain in this regard, including genetic heterogeneity challenges in delivering vectors to cardiac muscle, safety risks and off-target editing, ethical dilemmas surrounding pre-symptomatic treatment, and the high cost and scalability of gene therapy (151–155).

## Precision medicine and potential combination therapies

The introduction of myosin inhibitors has created an exciting future for HCM treatment. In general, HOCM management is beginning to mirror the wider shift in medicine towards precision care. There is an increasing use of genetic testing to help with risk stratification and reduce the likelihood of sudden cardiac death (156, 157). However, restricted access to genetic testing remains a reality in some health economies, including the UK (158). Artificial intelligence (AI) is increasingly being used to improve detection of HOCM in the population and may influence future treatment pathways (159). Furthermore, improvements in advanced imaging will help with early diagnosis and therefore gives more scope of early prophylactic treatment or management of symptoms. Artificial intelligence may also have a future role in genetic testing sensitivity and result interpretation to guide gene therapy options. Myosin inhibitors can potentially reduce or eliminate the need for invasive surgery, however their efficacy in gene positive vs. gene negative HOCM has not been established. There is also a role for dual therapy using medications and invasive interventions (160).

## Conclusion

The management of HOCM has evolved significantly in recent years. Historically, traditional pharmacotherapy with beta-blockers, calcium-channel blockers and disopyramide has provided symptomatic benefit, but many patients have required escalation to invasive septal reduction therapies. The development of myosin inhibition has introduced a new therapeutic class that is changing the escalation pathway in obstructive HCM and enabling clinicians to reassess when septal reduction therapy is required. Future treatment options may include revision of first-line therapy, combination therapy, and the exciting potential for gene therapy to further modify the disease process.
